# Hypertensive disorders of pregnancy and risk of diabetes in Indian women: a cross-sectional study

**DOI:** 10.1136/bmjopen-2015-011000

**Published:** 2016-08-05

**Authors:** Sutapa Agrawal, Jasmine Fledderjohann

**Affiliations:** 1 Centre for Chronic Conditions and Injuries, Public Health Foundation of India, New Delhi, India; 2 Department of Sociology, University of Oxford, Oxford, UK

**Keywords:** preeclampsia, eclampsia, diabetes, India, NFHS-3

## Abstract

**Background:**

Epidemiological data from high-income countries suggest that women with hypertensive disorders of pregnancy (HDP) are more likely to develop diabetes later in life.

**Objective:**

We investigated the association between pre-eclampsia and eclampsia (PE&E) during pregnancy and the risk of diabetes in Indian women.

**Design:**

Cross-sectional study.

**Setting:**

India.

**Methods:**

Data from India's third National Family Health Survey (NFHS-3, 2005–2006), a cross-sectional survey of women aged 15–49 years, are used. Self-reported symptoms suggestive of PE&E were obtained from 39 657 women who had a live birth in the 5 years preceding the survey. The association between PE&E and self-reported diabetes status was assessed using multivariable logistic regression models adjusting for dietary intake, body mass index (BMI), tobacco smoking, alcohol drinking, frequency of TV watching, sociodemographic characteristics and geographic region.

**Results:**

The prevalence of symptoms suggestive of PE&E in women with diabetes was 1.8% (n=207; 95% CI 1.5 to 2.0; p<0.0001) and 2.1% (n=85; 95% CI 1.8 to 2.3; p<0.0001), respectively, compared with 1.1% (n=304; 95% CI 1.0 to 1.4) and 1.2% (n=426; 95% CI 1.1 to 1.5) in women who did not report any PE&E symptoms. In the multivariable analysis, PE&E was associated with 1.6 times (OR=1.59; 95% CI 1.31 to 1.94; p<0.0001) and 1.4 times (OR=1.36; 95% CI 1.05 to 1.77; p=0.001) higher risk for self-reported diabetes even after controlling for dietary intake, BMI and sociodemographic characteristics.

**Conclusions:**

HDP is strongly associated with the risk of diabetes in a large nationally representative sample of Indian women. These findings are important for a country which is already tackling the burden of young onset of diabetes in the population. However, longitudinal medical histories and a clinical measurement of diabetes are needed in this low-resource setting.

**Strengths and limitations of this study**

- Epidemiological data from high-income countries suggests that women with hypertensive disorders of pregnancy (HDP) are more likely to develop cardiovascular risk, including diabetes, later in life. There is no empirical evidence of an association in low-income and middle-income countries, which have the highest burden.
- Our study provided strong evidence of an increased risk of diabetes among women reporting symptoms suggestive of pre-eclampsia or eclampsia during their last pregnancy. This positive relationship is robust even after adjustment for a comprehensive range of established risk factors for diabetes, possible clustering of lifestyles, other factors that may accompany HDP (eg, smoking, alcohol intake and access to healthcare), dietary predictors of diabetes (ie, food consumption), body mass index and education level.
- To the best of our knowledge, this is the largest nationally representative cross-sectional study of the population-based association between pregnancy-induced pre-eclampsia or eclampsia symptoms and diabetes risk in an Asian population.
- A history of pre-eclampsia or eclampsia during pregnancy should alert clinicians to the need for preventative counselling and more vigilant screening for diabetes.
- These findings are important for a country such as India, which is tackling the dual burden of noncommunicable diseases and infectious diseases among its female population.

## Introduction

Hypertensive disorders of pregnancy (HDP) represent a group of conditions marked by high blood pressure during pregnancy, proteinuria and, in some cases, convulsions. Gestational hypertension, pre-eclampsia and eclampsia (PE&E) are the most common HDP. The most serious consequences for the mother and the baby result from PE&E.^1^ Pre-eclampsia is a syndrome of pregnancy, defined by the onset of hypertension and proteinuria and characterised by widespread dysfunction of the endothelium in the mother.^2^ Eclampsia is usually a consequence of pre-eclampsia, and consists of central nervous system seizures unrelated to conditions such as epilepsy, which often leave the patient unconscious; if untreated, it may lead to death. Worldwide, HDP are more common, and may complicate 5–10% of all pregnancies. HDP are responsible for 12–25% of cases of maternal mortality during pregnancy and the puerperium.^3^ ^4^ PE&E are leading threats to safe motherhood in developing countries, where a woman is seven times more likely to develop these conditions.^5^ In such settings, it is estimated that 10–25% of these cases (an estimated ∼40 000 women) lead to maternal deaths annually.^5^

Increasing evidence indicates that PE&E is not just a disease of pregnancy that resolves at the time of delivery, but rather it represents a risk marker of cardiovascular diseases later in life.^6^ Studies in western populations examining the risk of developing type 2 diabetes in women with a history of pre-eclampsia have found a positive association equalling the risk attributed to obesity and smoking.^7–11^ The Danish National Patient Registry study, for example, found that pre-eclampsia is associated with a 3.1–3.7-fold risk of developing type 2 diabetes.^12^ Similarly, a recent population-based study of more than one million women found that women with pre-eclampsia or gestational hypertension have a twofold increased risk of developing diabetes after pregnancy.^13^

Studies showing the association between HDP and diabetes are generally based on non-representative clinical data and/or are based on data from western settings. Investigation of the links between PE&E symptoms and diabetes in low-income and middle-income countries (LMICs), which have the highest burden of these conditions, has been severely limited. Randomised trials have shown that diabetes can be prevented or delayed in high-risk groups by a variety of lifestyle and therapeutic interventions.^14^ ^15^ However, identifying at-risk populations to screen for diabetes in a low-resource setting such as India is a critical step in translating these findings into clinical practice. HDP such as pre-eclampsia may heighten the propensity for women to develop diabetes in the years following pregnancy; such women may also be suitable targets for diabetes prevention. We investigated the association between PE&E during pregnancy and diabetes risk in a large nationally representative sample of Indian women.

## Materials and methods

### Data

Data from the Indian National Family Health Survey (NFHS-3) for the years 2005/2006 are used in the study. The NFHS-3 is a large, well-established, nationally representative survey based on a multistage cluster random sampling design that provides high-quality information on the health and nutrition of women and children in India with an overall response rate of 98%.^16^ The NFHS is the Indian equivalent of the Demographic and Health Surveys, a standardised series of surveys routinely conducted in more than 80 developing countries. All data from these surveys are in the public domain and can be downloaded, after registration, from http://www.measuredhs.com. The NFHS has been conducted in India for three successive rounds, each at an interval of 5 years. The NFHS-3 collected demographic, socioeconomic and health information from a nationally representative probability sample of 124 385 women aged 15–49 years residing in 109 041 households. All states of India are represented in the sample (except the small Union Territories), covering more than 99% of the country's population. The survey was conducted using an interviewer-administered questionnaire in the native language of the respondent using a local, commonly understood term for any diseases. A total of 18 languages were used with back-translation to English to ensure accuracy and comparability. Full details of the survey have been published elsewhere.^16^

To examine the association between HDP and risk of diabetes, we restricted the sample to only those women who had a live birth in the 5 years preceding the survey. We further restricted our analyses to data pertaining to the most recent birth to minimise recall bias. Missing data were dropped using listwise deletion. This resulted in a final sample size of 39 657 participants for the analysis.

### Outcome evaluation

The survey includes self-report data relating to specific health problems of the mother, including whether the respondent currently has diabetes.^16^ Specifically, respondents were asked: ‘Do you currently have diabetes?’ with the response options of ‘yes’, ‘no’ and ‘don't know’. Notably, self-reported diabetes is not as accurate as clinical measures of diabetes as no physician diagnosis or fasting blood glucose measures were included in the NFHS-3.^16^

### Predictor variables

The NFHS-3 contains several items related to health problems during pregnancy. Since physical markers such as blood pressure, proteinuria) used in the clinical diagnosis of PE&E were not measured in the NFHS-3, we used three self-reported health items to construct a measure of PE&E. Specifically, in relation to their current or most recent pregnancy, mothers were asked: ‘During this pregnancy, did you have difficulty with your vision during daylight?’, ‘During this pregnancy, did you have swelling of the legs, body or face?’ and ‘During this pregnancy, did you have convulsions not from fever?’ The response options were ‘yes’, ‘no’ and ‘don't know’. Following the WHO^17^ and National Institute for Health and Care Excellence^18^ guidelines, and NFHS-3 coding by Agrawal *et al*,^19^ we created a dichotomous indicator for PE&E: women who reported both difficulty with vision during daylight and swelling of the legs, body or face were coded as having symptoms suggestive of pre-eclampsia, while those who additionally reported experiencing convulsions (not from fever) were coded as eclamptic.

### Covariates

In order to reduce the possibility that the association between PE&E and diabetes was driven by confounders, we included several sociodemographic control variables. Height and weight data were collected by the NFHS-3 interview staff, and body mass index (BMI; measured as kg/m^2^) was calculated based on these data.^16^ We used the standard Asian population cut-offs^20–22^ for the BMI measure, with thresholds defined as ≤18.4 kg/m^2^ (underweight), 18.5–22.9 kg/m^2^ (normal), 23.0–24.9 kg/m^2^ (overweight) and ≥25 kg/m^2^ (obese). Women who were pregnant at the time of the survey or women who had given birth during the 2 months preceding the survey were excluded from these measurements. In addition, previous studies have found that smokers are insulin resistant, exhibit several aspects of the insulin resistance syndrome and are at an increased risk for type 2 diabetes,^23^ while moderate alcohol consumption may reduce the risk of type 2 diabetes. On the other hand, binge drinking and high alcohol consumption may increase the risk of type 2 diabetes.^24^ We included controls for smoking and drinking behaviour: participants were asked four yes/no questions on current use of cigarettes, pipes, other local tobacco smoking products, and snuff, chew or other smokeless tobacco products. As a dichotomous measure of current tobacco use, we classified women as smokers if the response was ‘yes' to smoking cigarettes, pipes or other local smoking products. We constructed a dichotomous indicator of current alcohol use in the present analysis. Frequency of watching television (TV; almost every day, at least once weekly, less than once weekly, not at all), a categorical variable in the NFHS data, was used as a measure of sedentary behaviour. We measured access to healthcare with a categorical indicator of type of healthcare facility used (public medical sector, non-governmental organisation (NGO) trust hospital or clinic, private medical sector and other sources). Previous work^19^ has shown that iron intake and consuming a diversified diet is associated with a reduced risk of PE&E. Dietary intake as indicated by consumption of selected foods was assessed by asking, ‘How often do you yourself consume the following items: daily, weekly, occasionally or never?’ related to the consumption of milk or curd, pulses or beans, green leafy vegetables, other vegetables, fruits, eggs, and chicken, meat or fish.^16^

In order to reduce the risk of unobserved homogeneity in our models, we included a variety of sociodemographic controls. The sociodemographic factors considered in the present analysis included age in 5-year intervals (15–19, 20–24, 25–29, 30–34, 35–39, 40–44, 45–49); education, classified as no education, primary (5–7 years completed), secondary (8–9 years) or higher (10+ years); employment status (currently not working, working); religion (Hindu, Muslim, Christian, Sikh, others); caste/tribe (scheduled castes, scheduled tribes, other backward class, general); a standard wealth index compiled by the NFHS-3 (measured by an index based on household ownership of assets and graded as lowest, second, middle, fourth and highest); place of residence (urban, rural); and geographic regions of India (north, northeast, central, east, west, south).

### Statistical analysis

Descriptive statistics were calculated for all variables using standard methods. Differences in categorical variables were tested using Pearson's χ^2^ tests. Multivariable logistic regression analysis was used to estimate the effect of symptoms suggestive of PE&E on self-reported diabetes risk. In the first logistic regression model, we examined the unadjusted association between PE&E symptoms and diabetes risk independent of each other. In the second model, we adjusted for BMI status, tobacco smoking, alcohol drinking, frequency of TV watching and access to healthcare in order to assess how much of the variance in this association was explained by those factors. In the third model, we added food and micronutrient intake to our model. In the fourth and final model, we added sociodemographic characteristics in order to examine the association between PE&E symptoms and diabetes risk controlling for the confounders discussed above.

To adjust for the NFHS-3 sampling design, a sample weight was also included in the models.^11^ Results are presented as ORs with 95% CIs (OR; 95% CI). The estimation of CIs takes into account design effects due to clustering at the level of the primary sampling unit. Before carrying out the multivariate model, the possibility of multicollinearity between the covariates was assessed. In the correlation matrix of covariates, all pairwise Pearson correlation coefficients were found to be <0.5, suggesting that multicollinearity did not affect the findings. All analyses were conducted using the SPSS statistical software package V.19 (IBM SPSS Statistics, Chicago, Illinois, USA).

### Ethical considerations

The NFHS-3 survey received ethical approval from the International Institute for Population Science's Ethical Review Board and Indian Government. Prior informed written consent was obtained from each respondent. The analysis presented in this study is based on secondary analysis of existing survey data with all identifying information removed; no ethical approval was required.

## Results

Table 1 presents the descriptive statistics for the sample, and bivariate associations between diabetes and PE&E. Overall, 28.7% (n=11 361) women reported symptoms suggestive of pre-eclampsia during their last pregnancy, and 1 out of 10 women (10.3%; n=4071) reported symptoms suggestive of eclampsia. Thirty-eight per cent were underweight, while 15% were either overweight or obese. A very small number were current smokers (1.5%) or alcohol drinkers (2.3%), while 68% had access to the private medical sector to obtain their healthcare. One-third viewed TV almost every day. Most mothers (nearly 75%) were aged between 15 and 29 years, and almost half (47%) had no education. Seventy per cent were not employed at the time of the survey. A majority of the mothers (four out of five) were identified as Hindu, and two-fifths belonged to a scheduled caste. One-fourth belonged to households in the poorest wealth quintile. More than 70% of the mothers were residing in rural areas, while 28% were residents of Central India. Half of the mothers reported consuming milk/curd, and a majority reported consuming pulses/beans and vegetables on a daily or weekly basis. However, only one-third reported consuming fruit (34%) and eggs (30%) and only one-fourth or less consumed fish (27%) or chicken/meat (21%) on a daily or weekly basis.

**Table 1:** Sample distribution, number and distribution of diabetes cases and prevalence of diabetes according to PE&E and other factors among Indian women, 2005–2006

	Sample size	Diabetes cases		Diabetes prevalence (%)	χ^2^ p value
Characteristics	Per cent (N)	Reported (% (N))	Not reported (% (N))		
Total	39 612	512		1.3	
Pre-eclampsia symptoms					<0.0001
No	71.3 (28 250)	59.5 (304)	71.5 (27 933)	1.1	
Yes	28.7 (11 361)	40.5 (207)	28.5 (11 147)	1.8	
Eclampsia symptoms					<0.0001
No	89.7 (35 541)	83.4 (426)	89.8 (35 096)	1.2	
Yes	10.3 (4071)	16.6 (85)	10.2 (3984)	2.1	
Body mass index					0.023
Underweight (≤18.5 kg/m^2^)	38.0 (14 440)	41.8 (208)	38.0 (14 227)	1.4	
Normal (18.5–22.9 kg/m^2^)	46.9 (17 833)	40.6 (202)	47.0 (17 622)	1.1	
Overweight (23.0–24.9 kg/m^2^)	7.3 (2766)	9.4 (47)	7.3 (2720)	1.7	
Obese (≥25.0 kg/m^2^)	7.8 (2964)	8.2 (41)	7.8 (2919)	1.4	
Current tobacco smoking					0.259
No	98.5 (39 006)	98.0 (501)	98.5 (38 484)	1.3	
Yes	1.5 (606)	2.0 (10)	1.5 (596)	1.7	
Drinks alcohol					0.001
No	97.7 (38 690)	98.3 (488)	97.7 (38 184)	1.3	
Yes	2.3 (911)	4.7 (24)	2.3 (886)	2.6	
Frequency of TV viewing					0.001
Not at all	43.8 (17 351)	50.9 (260)	43.7 (17 076)	1.5	
Less than once a week	11.3 (449)	13.3 (68)	11.3 (4423)	1.5	
At least once a week	10.3 (4074)	9.0 (46)	10.3 (4027)	1.1	
Almost everyday	34.6 (13 689)	26.8 (137)	34.7 (13 548)	1.0	
Access to healthcare					<0.0001
Public medical sector	31.3 (11 313)	34.5 (162)	31.3 (11 138)	1.4	
NGO or trust or clinic	0.3 (113)	0.9 (4)	0.3 (109)	3.5	
Private medical sector	68.1 (24 591)	63.4 (298)	68.1 (24 279)	1.2	
Other source	0.3 (119)	1.3 (6)	0.3 (112)	5.1	
*Food and micronutrient intake*					
Diversified dietary intake					0.193
Inadequate	68.9 (27 275)	68.8 (26 895)	70.7 (362)	1.3	
Adequate	31.1 (12 337)	31.2 (12 185)	29.3 (150)	1.2	
Intake of iron and folic acid supplementation					0.212
No	74.7 (29 588)	74.7 (29 183)	76.3 (390)	1.3	
Yes	25.3 (10 024)	25.3 (9897)	23.7 (121)	1.2	
Milk or curd					<0.0001
Never/occasionally	48.1 (19 046)	56.8 (290)	48.0 (18 745)	1.5	
Daily/weekly	51.9 (20 561)	43.2 (221)	52.0 (20 331)	1.1	
Pulses or beans					<0.0001
Never/occasionally	10.1 (4014)	10.1 (3935)	10.1 (3935)	1.9	
Daily/weekly	89.9 (35 588)	89.9 (35 137)	89.9 (35 137)	1.2	
Vegetables					0.075
Never/occasionally	7.1 (2804)	7.1 (2759)	7.1 (2759)	1.6	
Daily/weekly	92.9 (36 795)	92.9 (36 310)	92.9 (36 310)	1.3	
Fruits					0.002
Never/occasionally	65.7 (26 043)	72.0 (368)	65.7 (25 663)	1.4	
Daily/weekly	34.2 (13 541)	28.0 (143)	34.3 (13 390)	1.1	
Eggs					0.084
Never/occasionally	69.7 (27 601)	72.6 (371)	69.7 (27 216)	1.3	
Daily/weekly	30.3 (11 985)	27.4 (140)	30.3 (11 841)	1.2	
Fish					0.003
Never/occasionally	73.3 (29 043)	67.9 (347)	73.4 (28 679)	1.2	
Daily/weekly	26.7 (10 558)	32.1 (164)	26.6 (10 392)	1.6	
Chicken/meat					0.004
Never/occasionally	78.7 (31 158)	83.6 (428)	78.6 (30 714)	1.4	
Daily/weekly	21.3 (8439)	16.4 (84)	21.4 (8352)	1.0	
*Sociodemographic characteristics*					
Age groups					0.001
15–19	7.5 (2982)	10.2 (52)	7.5 (2928)	1.7	
20–24	33.5 (13 269)	29.0 (148)	33.6 (13 113)	1.1	
25–29	32.6 (12 908)	27.2 (139)	32.7 (12 767)	1.1	
30–34	16.9 (6685)	18.8 (96)	16.8 (6584)	1.4	
35–39	6.9 (2723)	11.0 (56)	6.8 (2667)	2.1	
40–44	2.1 (835)	2.7 (14)	2.1 (818)	1.7	
45–49	0.5 (210)	1.2 (6)	0.5 (204)	2.9	
Education					0.064
No education	18 758 (47.4)	51.4 (263)	47.3 (18 480)	1.4	
Primary	5545 (14.0)	14.6 (75)	14.0 (5470)	1.4	
Secondary	12 947 (32.7)	30.3 (155)	32.7 (12 790)	1.2	
Higher	2361 (6.0)	3.7 (19)	6.0 (2339)	0.8	
Employment status					0.143
Currently not working	27 665 (69.9)	67.7 (346)	70.0 (27 309)	1.3	
Working	11 886 (30.1)	32.3 (165)	30.0 (11 713)	1.4	
Religion					0.035
Hindu	31 248 (78.9)	76.8 (393)	78.9 (30 836)	1.3	
Muslim	6472 (16.3)	17.2 (88)	16.3 (6383)	1.4	
Christian	811 (2.0)	3.9 (20)	2.0 (791)	2.5	
Sikh	513 (1.3)	0.8 (4)	1.3 (509)	0.8	
Others	568 (1.4)	1.4 (7)	1.4 (561)	1.2	
Caste/tribe					<0.0001
Scheduled caste	7938 (20.1)	21.3 (109)	20.1 (7825)	1.4	
Scheduled tribes	3740 (9.4)	13.7 (70)	9.4 (3666)	1.9	
Other backward class	15 861 (40.2)	30.3 (155)	40.3 (15 696)	1.0	
General	10 830 (27.4)	30.9 (158)	27.4 (10 669)	1.5	
Missing caste	1085 (2.8)	3.7 (19)	2.7 (1060)	1.8	
Wealth index					<0.0001
Lowest	9553 (24.1)	32.4 (166)	24.0 (9381)	1.7	
Second	8588 (21.7)	22.3 (114)	21.7 (8465)	1.3	
Middle	7762 (19.6)	19.9 (102)	19.6 (7661)	1.3	
Fourth	7251 (18.3)	15.6 (80)	18.3 (7168)	1.1	
Highest	6458 (16.3)	9.8 (50)	16.4 (6405)	0.8	
Place of residence					0.001
Urban	10 615 (26.8)	20.3 (104)	26.9 (10 506)	1.0	
Rural	28 997 (73.2)	79.7 (408)	73.1 (28 574)	1.4	
Geographic regions					<0.0001
North	5076 (12.8)	9.4 (48)	12.9 (5028)	0.9	
Northeast	1607 (4.1)	5.9 (30)	4.0 (1576)	1.9	
Central	11 099 (28.0)	17.8 (91)	28.4 (11 000)	0.8	
East	10 031 (25.3)	48.3 (247)	25.0 (9777)	3.5	
West	5114 (12.9)	6.1 (31)	13.0 (5081)	0.6	
South	6684 (16.9)	12.5 (64)	16.9 (6618)	1.0	

Number of women varies slightly for individual variables depending on the number of missing values.

NGO, non-governmental organisation; PE&E, pre-eclampsia and eclampsia.

Of the women reporting diabetes, two out of five (41%) also reported symptoms suggestive of pre-eclampsia, and 17% reported symptoms of eclampsia (table 1). Eighteen per cent were either obese or overweight; 2% were currently smoking tobacco; 5% were alcohol drinkers; half reported not watching TV at all; one in three reported visiting the public medical sector for healthcare needs; two-thirds (66%) were in the age group 15–29 years; half of them had no education; 77% were Hindus; 31% belonged to the general category; 68% were not working; two-fifths belonged to households in the poorest wealth quintile; a majority resided in a rural area and half resided in eastern India. Among the women who reported diabetes, a majority of them reportedly consumed pulses/beans and vegetables on a daily/weekly basis and more than two-fifth also reported consuming milk/curd daily/weekly, while consumption of fruits (28%), eggs (27%), fish (32%) or chicken meat (16%) was less frequent.

The prevalence of symptoms suggestive of PE&E in women with diabetes was 1.8% (95% CI 1.5% to 2.0%; p<0.0001) and 2.1% (95% CI 1.8% to 2.3%; p<0.0001), respectively, compared with 1.1% (95% CI 1.0% to 1.4%) and 1.2% (95% CI 1.1% to 1.5%) in women who did not report any PE&E symptoms (table 1). Overweight (1.7%) or obese (1.4%) women had a higher prevalence of diabetes than those who were underweight (1.4%) and of normal weight (1.1%). A higher proportion of current tobacco smokers (1.7%) and current alcohol drinkers (2.6%) also reported diabetes compared with those who did not engage in these risky behaviours. Compared with those using public sector facilities, women who had access only to other sources for healthcare (5.1%) and NGO or trust or clinic (3.5) had a higher prevalence of diabetes. Women reporting viewing TV not at all or less than once a week reported a higher prevalence of diabetes (1.5%) than more frequent viewers. Women consuming milk/curd (1.1% vs 1.5%), pulses/beans (1.2% vs 1.9%), vegetables (1.3% vs 1.6%), fruits (1.1% vs 1.4%), eggs (1.2% vs 1.3%) or chicken/meat (1.0% vs 1.4%) except fish (1.6% vs 1.2%) on a daily/weekly basis had a lower prevalence of diabetes than those who never or occasionally consumed them. Diabetes prevalence was higher among women aged 40–49 (2.9%) and 35–39 (2.1%) years compared with women aged 15–29 years (1.2%). The prevalence of diabetes was also higher among women belonging to the Christian religion (2.5%), among women belonging to a scheduled tribe (1.9%) compared with those in a scheduled caste (1.4%), women in the poorest wealth quintile (1.7%), women residing in rural areas (1.4%) and women living in eastern India (3.5%).

Table 2 shows the results of multivariable logistic regression analyses in unadjusted, partially adjusted and fully adjusted models. In the unadjusted analysis (model 1), the likelihood of having diabetes was significantly higher among women who reported pre-eclampsia (OR 1.71; 95% CI 1.43 to 2.04; p<0.0001) and eclampsia (OR 1.76; 95% CI 1.40 to 2.23; p<0.0001) symptoms than among those who did not report these symptoms. Controlling for BMI, tobacco smoking, alcohol intake, TV watching and healthcare access (in model 2) slightly attenuated the positive relationship between pre-eclampsia (OR 1.62; 95% CI 1.33 to 1.96; p<0.0001) and eclampsia (OR 1.48; 95% CI 1.15 to 1.91; p=0.003) symptoms and diabetes, but the association remained positive, strong and significant. The positive association between pre-eclampsia (OR 1.59; 95% CI 1.31 to 1.93; p<0.0001) or eclampsia (OR 1.50; 95% CI 1.16 to 1.95; p=0.002) and diabetes remained virtually unchanged when specific food and micronutrient intakes were additionally controlled for in model 3. The final model (model 4) in table 2 provides the fully adjusted analysis with all covariates included. Jointly controlling for all of these factors, the positive association between symptoms suggestive of pre-eclampsia (OR 1.59; 95% CI 1.31 to 1.94; p<0.0001) or eclampsia (OR 1.36; 95% CI 1.05 to 1.77; p=0.020) during pregnancy and diabetes remained strong and statistically significant.

**Table 2:** Unadjusted and partially adjusted and fully adjusted ORs and 95% CI showing the association between PE&E and other factors and diabetes risk among Indian women, 2005–2006

	Unadjusted model 1	Adjusted model 2	Adjusted model 3	Adjusted model 4
	OR (95% CI)	OR (95% CI)	OR (95% CI)	OR (95% CI)
Pre-eclampsia symptoms				
No^Ref^	1	1	1	1
Yes	1.71 (1.43 to 2.04)	1.62 (1.33 to 1.96)	1.59 (1.31 to 1.93)	1.59 (1.31 to 1.94)
Eclampsia symptoms				
No^Ref^	1	1	1	1
Yes	1.76 (1.40 to 2.23)	1.48 (1.15 to 1.91)	1.50 (1.16 to 1.95)	1.36 (1.05 to 1.77)
BMI				
Underweight (≤18.5 kg/m^2^)		1.24 (1.01 to 1.52)	1.21 (0.99 to 1.49)	1.17 (0.95 to 1.44)
Normal (18.5–22.9 kg/m^2^)^Ref^		1	1	1
Overweight (23.0–24.9 kg/m^2^)		1.74 (1.25 to 2.42)	1.78 (1.27 to 2.47)	2.01 (1.43 to 2.81)
Obese (≥25.0 kg/m^2^)		1.47 (1.02 to 2.10)	1.52 (1.06 to 2.19)	1.85 (1.26 to 2.72)
Current tobacco smoking				
No^Ref^		1	1	1
Yes		1.13 (0.60 to 2.11)	1.13 (0.60 to 2.12)	0.88 (0.46 to 1.68)
Current alcohol drinking				
No^Ref^		1	1	1
Yes		1.93 (1.26 to 2.95)	1.81 (1.17 to 2.79)	1.36 (0.84 to 2.20)
Frequency of TV viewing				
Not at all^Ref^		1	1	1
Less than once a week		1.04 (0.78 to 1.38)	1.03 (0.77 to 1.38)	1.21 (0.90 to 1.63)
At least once a week		0.81 (0.58 to 1.13)	0.84 (0.60 to 1.18)	1.02 (0.72 to 1.46)
Almost everyday		0.72 (0.57 to 0.90)	0.80 (0.62 to 1.02)	1.18 (0.87 to 1.61)
Access to healthcare				
Public medical sector^Ref^		1	1	1
NGO or trust or clinic		2.03 (0.71 to 5.76)	2.06 (0.72 to 5.91)	1.76 (0.60 to 5.18)
Private medical sector		0.84 (0.69 to 1.02)	0.89 (0.73 to 1.08)	0.89 (0.72 to 1.10)
Other source		3.16 (1.31 to 7.59)	3.08 (1.27 to 7.44)	2.57 (1.05 to 6.33)
*Food and micronutrient intake*				
Diversified dietary intake				
Inadequate^Ref^			1	1
Adequate			1.54 (1.16 to 2.04)	1.46 (1.10 to 1.94)
Intake of iron and folic acid supplementation				
No^Ref^			1	1
Yes			1.20 (0.81 to 1.29)	1.11 (0.87 to 1.41)
Milk or curd				
Never/occasionally^Ref^			1	1
Daily/weekly			0.87 (0.71 to 1.07)	1.06 (0.85 to 1.31)
Pulses or beans				
Never/occasionally^Ref^			1	1
Daily/weekly			0.76 (0.58 to 1.00)	0.80 (0.60 to 1.07)
Vegetables				
Never/occasionally^Ref^			1	1
Daily/weekly			0.90 (0.65 to 1.25)	0.81 (0.58 to 1.13)
Fruits				
Never/occasionally^Ref^			1	1
Daily/weekly			0.92 (0.73 to 1.15)	1.05 (0.82 to 1.33)
Eggs				
Never/occasionally^Ref^			1	1
Daily/weekly			0.90 (0.70 to 1.16)	0.80 (0.62 to 1.03)
Fish				
Never/occasionally^Ref^			1	1
Daily/weekly			1.76 (1.38 to 2.25)	1.12 (0.87 to 1.46)
Chicken/meat				
Never/occasionally^Ref^			1	1
Daily/weekly			0.60 (0.45 to 0.80)	0.75 (0.56 to 1.02)
*Socioeconomic and demographic characteristics*				
Age				
15–19^Ref^				1
20–24				0.83 (0.58 to 1.18)
25–29				0.71 (0.49 to 1.02)
30–34				0.86 (0.58 to 1.27)
35–39				1.25 (0.81 to 1.91)
40–44				0.99 (0.53 to 1.86)
45–49				1.49 (0.60 to 3.68)
Education				
No education^Ref^				1
Primary				0.92 (0.68 to 1.25)
Secondary				1.15 (0.87 to 1.52)
Higher				0.96 (0.53 to 1.74)
Employment status				
Currently not working^Ref^				1
Working				1.21 (0.91 to 1.38)
Religion				
Hindu^Ref^				1
Muslim				1.00 (0.74 to 1.35)
Christian				1.53 (0.90 to 2.60)
Sikh				0.79 (0.27 to 2.31)
Others				0.65 (0.29 to 1.47)
Caste/tribe				
Scheduled caste^Ref^				1
Scheduled tribes				0.97 (0.68 to 1.38)
Other backward class				0.65 (0.50 to 0.85)
General				1.11 (0.83 to 1.49)
Missing caste				0.48 (0.24 to 0.95)
Wealth index				
Lowest^Ref^				1
Second				0.89 (0.68 to 1.16)
Middle				0.80 (0.58 to 1.09)
Fourth				0.76 (0.52 to 1.11)
Highest				0.45 (0.27 to 0.78)
Place of residence				
Urban^Ref^				1
Rural				1.01 (0.77 to 1.34)
Geographic regions				
North^Ref^				1
Northeast				1.82 (1.06 to 3.13)
Central				0.93 (0.62 to 1.39)
East				2.68 (1.83 to 3.93)
West				0.69 (0.42 to 1.15)
South				1.28 (0.82 to 1.99)
Number of cases				34 978

Note: ^Ref^ denotes reference category; model 1 unadjusted; model 2 adjusted for BMI, tobacco smoking, alcohol drinking, TV watching and access to healthcare; Model 3 adjusted for model 2+specific dietary intakes; model 4 adjusted for all.

BMI, body mass index; NGO, non-governmental organisation; PE&E, pre-eclampsia and eclampsia; TV, television.

As shown in the full model (model 4) in table 2, with all other variables controlled for, being overweight (OR 2.01; 95% CI 1.43 to 2.81; p=0.030) or obese (OR 1.85; 95% CI 1.26 to 2.72; p=0.010) had a positive and statistically significant effect on risk of diabetes among women. Women seeking healthcare from other sources had 2.5 times higher odds of diabetes (OR 2.57; 95% CI 1.05 to 6.33; p=0.015) compared with those accessing healthcare through the public sector. Women consuming an adequately diversified diet also had a higher likelihood of reporting diabetes (OR 1.46; 95% CI 1.10 to 1.94; p=0.005) than women consuming an inadequately diversified diet. Women residing in the northern (OR 1.82; 95% CI 1.06 to 3.13; p=0.005) and eastern regions of India (OR 2.68; 95% CI 1.83 to 3.93; p=0.001) also had a higher likelihood of reporting diabetes risk than women residing in other parts of India. The remaining covariates were not significantly associated with odds of reporting diabetes.

## Discussion

In this study, we examined the association between HDP, focusing specifically on PE&E, and diabetes risk in a large, nationally representative sample of Indian women. We observed strong evidence of an increased risk of diabetes among women reporting symptoms suggestive of PE&E during their last pregnancy. This association is robust, as we have adjusted for a comprehensive range of established risk factors of diabetes and possible clustering of lifestyles and other factors that may accompany HDP (eg, smoking, alcohol intake, access to healthcare) and dietary predictors of diabetes (ie, food consumption), BMI and education level. Interestingly, we found evidence of an association between diabetes and lifestyle factors (eg, watching TV), as well as evidence of regional variation in the prevalence of diabetes. However, many of these associations were attenuated or non-significant in the adjusted models, highlighting the importance of the sociodemographic patterning of lifestyle and health behaviours.

To the best of our knowledge, this is the largest nationally representative cross-sectional study at the population level showing the association between PE&E and diabetes risk in an Asian population. Another strength of this study is our ability to adjust for obesity, which in itself is associated with insulin resistance, and is a well-known risk factor for the development of diabetes and pre-eclampsia. These findings highlight and support the need to counsel patients with hypertensive disorders of pregnancy regarding postpartum diabetes screening and prevention in a developing country setting.

The long-term sequelae of both PE&E are not well evaluated in LMICs. This lack of evidence is particularly problematic in India, where the rate of HDP-related maternal mortality is high,^1^ and PE&E are thought to underlie around 5–10% of pregnancy complications and about 8–9% of maternal deaths in India.^25^ Some clinical studies suggest that the proportion of deliveries impacted by PE&E in Indian women ranges from as low as 0.9% to as high as 7.7% of all deliveries.^25^ However, these clinical studies are likely to suffer from selection bias on the basis of severity of the condition, especially among populations with limited access to prenatal care, and therefore may underestimate the prevalence of the condition.

Common pathogenic pathways may underlie the association between women with a history of pre-eclampsia and an increased risk of diabetes as each of these conditions is associated with manifestations of metabolic syndrome (including endothelial dysfunction, obesity, hypertension, hyperglycaemia, insulin resistance, renal disease and dyslipidaemia). Metabolic syndrome is known for its association with insulin resistance during pregnancy,^26–28^ which may be independent of obesity and glucose intolerance.^26^ ^27^ ^29^ These conditions may subsequently predispose women to develop hypertension, atherosclerosis and type 2 diabetes mellitus in later life, which eventually lead to cardiovascular disease.^10^ ^11^ ^30^ Other possible explanations for this association include the following: (1) since both cardiovascular disease and pre-eclampsia share common risk factors, pregnancy serves as a ‘stress test’, with the development of HDP identifying a woman at high risk of developing cardiovascular disease later in life;^31^ (2) pregnancy, and especially PE&E, may induce permanent arterial changes; the proatherogenic stress of pregnancy, often excessive in women with pre-eclampsia, could activate arterial wall inflammation that fails to resolve after delivery, increasing the risk of future cardiovascular disease.^11^ Women with early onset/severe pre-eclampsia, recurrent pre-eclampsia or pre-eclampsia with onset as a multipara appear to face the highest risk of cardiovascular disease later in life, including during the premenopausal period.^6^

### Limitations of the study

There are several limitations to our study. First, most variables in the analyses (with the exception of anthropometrics) were self-reported, including a symptomatic rather than clinical measure of pre-eclampsia and diabetes. It is possible that self-reported data may suffer from recall bias. Moreover, many pregnant women may experience oedema that is not symptomatic of pre-eclampsia, and vision difficulties may be indicative of pre-eclampsia, as well as secondary to gestational diabetes. Although we cannot rule out the possibility of misclassification within this context, it is unlikely that we have missed severe PE&E or diabetes cases due to the generally clear manifestation of symptoms in severe cases. However, mild cases of pre-eclampsia may be asymptomatic, and would most likely be missed by our measure here. This possibility limits the applicability of our findings, as the majority of patients in clinical practice have mild-to-moderate pre-eclampsia, and may comprise much of this asymptomatic group. The presence or absence of convulsions may have greater face validity as a measure of eclampsia (as compared with swelling and blurred vision for pre-eclampsia), and we thus have greater confidence in our findings for eclampsia. Notably, however, we included PE&E as separate measures in the models, but found the coefficients for these measures to be in the same direction and of similar magnitude, providing some confidence in the validity of our measure of pre-eclampsia as well.

Second, owing to the nature of the data, we could not identify precise timing during the gestational period of pre-eclampsia symptoms, nor the precise onset of diabetes. However, the majority of individuals with type 2 diabetes mellitus have no symptoms and can be misclassified as patients without diabetes in the self-reports. This error may in fact strengthen our findings. Furthermore, family history, physical activity, glucose and blood pressure measures are also known risk factors for diabetes, which were not collected in the survey. From the data used here, we could not differentiate type 1 from type 2 diabetes; however, given that the mean age of the women was 26.4 years (±5.6 SD), it is most likely that the majority of the women developed type 2 diabetes. We were, however, able to adjust for several other important confounding variables including socioeconomic and demographic factors, lifestyle indicators and access to healthcare.

## Conclusions

In summary, this study provides some initial empirical evidence that HDP, specifically symptoms suggestive of PE&E during pregnancy, are strongly associated with diabetes in a large nationally representative sample of Indian women. This initial evidence provides an impetus for further evaluation. These findings have important implications for maternal and child health, especially given the increase in obesity-related diseases in this low-resource setting. A history of PE&E during pregnancy should alert clinicians to the need for preventative counselling and more vigilant screening for diabetes, and women should be encouraged to have a more rigorous follow-up and adopt a healthier lifestyle. Indeed, lifestyle factors were significantly associated with diabetes in our study (eg, TV viewing, dietary patterns). While these associations were attenuated by the inclusion of sociodemographic controls, this attenuation most likely points to the socioeconomic gradient in lifestyle and health behaviours. Follow-up and counselling of women with a history of PE&E may offer a window of opportunity for prevention of future diabetes mellitus and cardiovascular disease. Patient and healthcare provider education is also essential for the successful assessment and management of cardiovascular disease risk and prevention in the context of the long-term burden associated with PE&E. Clinical awareness of a history of PE&E might allow the identification of cases not previously recognised as at risk for diabetes, facilitating the implementation of preventive measures. Moreover, evaluation of women prior to pregnancy and follow-up during pregnancy is needed to determine the role of shared risk factors. Regular medical follow-up and earlier screening for cardiovascular disease should be considered in this population. At the least, current screening guidelines should be followed, and women should receive advice on established preventive lifestyle measures and treatment strategies that should be implemented by all women, regardless of a previous history of gestational hypertension/pre-eclampsia.^32^ Further research to verify accuracy of reporting of the symptoms of PE&E is needed, and would be facilitated by longitudinal medical histories and a clinical measurement of diabetes in an Indian setting. Additional work is needed to investigate the association between diabetes and mild pre-eclampsia, particularly asymptomatic cases that may be picked up in a clinical setting.
